# Research hotspots in pediatrics: co-word clustering analysis based on readership in PubMed Central

**DOI:** 10.3389/fped.2024.1460954

**Published:** 2024-10-16

**Authors:** Fangming Deng, Wen Sun, Jiangwei Guo, Yujia Yang

**Affiliations:** ^1^ Editorial Office of Chinese Journal of Contemporary Pediatrics, Xiangya Medical Academic Promotion Center, Xiangya Hospital, Central South University, Changsha, China; ^2^ IDMED Research Lab, Beijing Intelligent Decision Medical Technology Co., Ltd., Beijing, China; ^3^ Xiangya Medical Academic Promotion Center, Xiangya Hospital, Central South University, Changsha, China

**Keywords:** PubMed Central, pediatrics, research hotspots, word frequency analysis, co-word analysis

## Abstract

**Objective:**

By analyzing high readership articles from the *Chinese Journal of Contemporary Pediatrics* (*CJCP*) in the PubMed Central (PMC) database, this study aims to identify research hotspots and trends in the field of pediatrics.

**Methods:**

Articles from the *CJCP* ranked by annual readership in PMC from 2021 to 2023 were collected. Using word frequency analysis and co-word analysis, the thematic characteristics of these articles were explored.

**Results:**

The word frequency analysis and co-word analysis revealed four thematic directions that were of significant interest to researchers: (1) current public health or medical events such as COVID-19 and influenza; (2) mental health issues in children and adolescents; (3) pediatric neurological diseases and neurodevelopment; (4) diseases in preterm infants and newborns.

**Conclusions:**

This study provides pediatric researchers with a valuable perspective to understand and grasp the development dynamics and future directions in the field of pediatrics.

## Introduction

In an increasingly globalized context, the integration of research and practice in pediatrics has led to significant scientific advancements and clinical insights. The *Chinese Journal of Contemporary Pediatrics* (*CJCP*) has emerged as a leading platform for disseminating these developments, gaining recognition for its high-quality academic content and practical relevance to pediatric clinicians and researchers worldwide. Since being indexed in MEDLINE (PubMed) in January 2006 and subsequently in PubMed Central (PMC) in August 2020, *CJCP*'s academic stature has grown, with its influence reflected in readership metrics. Currently, the journal receives over 70,000 monthly reads in the PMC database—managed by the National Center for Biotechnology Information in the U.S.—which serves as a crucial resource for free access to biomedical literature.

Despite the vast amount of academic information available, pediatric researchers face challenges in identifying research hotspots and trends. In this era of networked information sharing, the number of downloads and reads post-publication is a valuable indicator of research interest (1). This study analyzes article readership from the *CJCP* in PMC between 2021 and 2023, employing word frequency and co-word analysis methods to uncover high-readership articles. By systematically sorting and analyzing these articles, we aim to identify key focal points and emerging trends in pediatric research, offering vital references for researchers navigating the evolving landscape of pediatrics.

We now present a detailed analysis of *CJCP*'s high-readership articles from 2021 to 2023.

## Methods

### Data sources

Data for this study were sourced from the PMC Publisher Statistics platform (https://www.ncbi.nlm.nih.gov/pmc/publisherservices/), which provides network backend access to journal publishers. We extracted the top 25 articles by annual readership from the *CJCP* publications between 2016 and 2023 for the years 2021 to 2023 (Tables 1–3). The *CJCP* published a total of 1,784 articles from 2016 to 2023, all of which are available for free reading on PMC. These articles include the number of publications for each year from 2016 to 2023: 252, 246, 204, 226, 232, 208, 216, and 200 articles, respectively.

**Table 1:** Top 25 most read articles in the *CJCP* on PMC in 2021 (descending order**).**

No.	Title	PMC number	Read frequency
1	Efficacy and safety of COVID-19 vaccines: a systematic review	PMC7969187	13,723
2	Penis growth and development in children and adolescents: a study based on GAMLSS	PMC7390054	8,314
3	Kawasaki disease—a new manifestation of COVID-19 in children	PMC7389629	6,439
4	Clinical features of children with SARS-CoV-2 infection: an analysis of 115 cases	PMC7389688	5,688
5	A review on the management of tic disorders in children: psychoeducation and behavioral intervention	PMC7389019	3,157
6	Effects of virtual reality training on limb movement in children with spastic diplegia cerebral palsy	PMC7389551	2,304
7	Clinical features of asymptomatic or subclinical COVID-19 in children	PMC7390219	1,866
8	A review on neonatal acute respiratory distress syndrome	PMC7389165	1,854
9	Roles of the public-facility-turned temporary hospital in prevention and control of coronavirus disease 2019 in Wuhan, China and clinical experience in the hospital	PMC7389389	1,810
10	Optimizing nutrition of the preterm infant	PMC7390124	1,636
11	Features and management of very early onset inflammatory bowel disease	PMC7389054	1,625
12	Clinical guidelines for the diagnosis and treatment of neonatal necrotizing enterocolitis (2020)	PMC7818154	1,571
13	Advances in research on childhood neutropenia	PMC7389658	1,530
14	Clinical characteristics of urticaria in children vs. adults	PMC7389927	1,525
15	Group B streptococcus colonization in pregnant women and group B streptococcus infection in their preterm infants	PMC7389577	1,503
16	Etiology and genetic diagnosis of short stature in children	PMC7389227	1,449
17	Effect of advanced maternal age on birth defects and postnatal complications of neonates	PMC7389863	1,434
18	Research advances in the pathogenesis and treatment of neurodegeneration with brain iron accumulation	PMC8213993	1,351
19	Effect of suspension exercise training on motor and balance functions in children with spastic cerebral palsy	PMC7389949	1,346
20	Some thoughts on influenza vaccine and regular influenza vaccination for healthcare workers	PMC7389024	1,311
21	A review on the prevention and treatment of congenital cytomegalovirus infection in mothers and infants	PMC7389047	1,294
22	Association of suicidal ideation with family environment and psychological resilience in adolescents	PMC7389414	1,260
23	Clinical guidelines for the diagnosis and treatment of feeding intolerance in preterm infants (2020)	PMC7568993	1,049
24	Advances in application of Jurkat cell model in research on infectious diseases	PMC7389782	993
25	Response plan in the neonatal intensive care unit during epidemic of SARS-CoV-2 infection (2nd Edition)	PMC7389594	984

**Table 2:** Top 25 most read articles in the *CJCP* on PMC in 2022 (descending order**).**

No.	Title	PMC number	Read frequency
1	Efficacy and safety of COVID-19 vaccines: a systematic review	PMC7969187	8,931
2	Kawasaki disease—a new manifestation of COVID-19 in children	PMC7389629	8,690
3	Penis growth and development in children and adolescents: a study based on GAMLSS	PMC7390054	7,656
4	Strengthening the prevention and treatment of Omicron infection in children	PMC9044981	7,343
5	Clinical features of asymptomatic or subclinical COVID-19 in children	PMC7390219	7,300
6	Influence of coronavirus disease 2019 on the nervous system of children	PMC8140346	6,804
7	Epidemiological and clinical features of children with mild coronavirus disease 2019	PMC8140334	5,857
8	Selective bronchial intubation for one-lung ventilation and independent-lung ventilation in pediatric age: state of the art	PMC7390223	5,002
9	A review on the management of tic disorders in children: psychoeducation and behavioral intervention	PMC7389019	4,072
10	Clinical features of children with SARS-CoV-2 infection: an analysis of 115 cases	PMC7389688	3,815
11	Influencing factors for duration of viral nucleic acid shedding in children with influenza A	PMC7403092	3,554
12	Association of depression and suicidal ideation with parenting style in adolescents	PMC8480172	3,478
13	Effect of advanced maternal age on birth defects and postnatal complications of neonates	PMC7389863	3,452
14	Research advances in the pathogenesis and treatment of neurodegeneration with brain iron accumulation	PMC8213993	3,438
15	Recent research on the treatment of spinal muscular atrophy	PMC8884051	3,151
16	Association of suicidal ideation with family environment and psychological resilience in adolescents	PMC7389414	3,110
17	Roles of the public-facility-turned temporary hospital in prevention and control of coronavirus disease 2019 in Wuhan, China and clinical experience in the hospital	PMC7389389	2,909
18	Clinical guidelines for the diagnosis and treatment of neonatal necrotizing enterocolitis (2020)	PMC7818154	2,812
19	Some thoughts on influenza vaccine and regular influenza vaccination for healthcare workers	PMC7389024	2,685
20	A retrospective analysis of medication in children with SARS-CoV-2 infection in Wuhan, China	PMC7818160	2,575
21	Features and management of very early onset inflammatory bowel disease	PMC7389054	2,271
22	An epidemiological study on human rhinovirus C in hospitalized children with respiratory tract infections	PMC7389860	2,237
23	A review on neonatal acute respiratory distress syndrome	PMC7389165	2,074
24	Effect of suspension exercise training on motor and balance functions in children with spastic cerebral palsy	PMC7389949	1,856
25	A review on the prevention and treatment of congenital cytomegalovirus infection in mothers and infants	PMC7389047	1,855

**Table 3:** Top 25 most read articles in the *CJCP* on PMC in 2023 (descending order**).**

No.	Title	PMC number	Read frequency
1	Influencing factors for duration of viral nucleic acid shedding in children with influenza A	PMC7403092	22,978
2	Penis growth and development in children and adolescents: a study based on GAMLSS	PMC7390054	12,044
3	Strengthening the prevention and treatment of Omicron infection in children	PMC9044981	6,962
4	A review on the management of tic disorders in children: psychoeducation and behavioral intervention	PMC7389019	5,359
5	Kawasaki disease—a new manifestation of COVID-19 in children	PMC7389629	4,788
6	Association of depression and suicidal ideation with parenting style in adolescents	PMC8480172	4,651
7	Recent research on the treatment of spinal muscular atrophy	PMC8884051	4,145
8	Research advances in the pathogenesis and treatment of neurodegeneration with brain iron accumulation	PMC8213993	3,894
9	Clinical features of Mycoplasma pneumoniae pneumonia with adenovirus infection in children	PMC8549638	3,859
10	Efficacy and safety of COVID-19 vaccines: a systematic review	PMC7969187	3,298
11	Clinical features of asymptomatic or subclinical COVID-19 in children	PMC7390219	3,114
12	Epidemiological and clinical features of children with mild coronavirus disease 2019	PMC8140334	2,962
13	Selective bronchial intubation for one-lung ventilation and independent-lung ventilation in pediatric age: state of the art	PMC7390223	2,832
14	Influence of coronavirus disease 2019 on the nervous system of children	PMC8140346	2,819
15	Clinical guidelines for the diagnosis and treatment of neonatal necrotizing enterocolitis (2020)	PMC7818154	2,605
16	An epidemiological study on human rhinovirus C in hospitalized children with respiratory tract infections	PMC7389860	2,548
17	Neuropsychological development of late preterm infants and early term infants at the age of 1 year: a follow-up study	PMC7389612	2,501
18	A clinical study of influenza A virus infection with neurological symptoms in children	PMC8140337	2,501
19	A retrospective analysis of medication in children with SARS-CoV-2 infection in Wuhan, China	PMC7818160	2,410
20	Risk factors for poor prognosis in children with severe adenovirus pneumonia	PMC7389467	2,317
21	Features and management of very early onset inflammatory bowel disease	PMC7389054	2,043
22	Risk factors for preterm birth: a prospective cohort study	PMC8690713	2,043
23	Effect of suspension exercise training on motor and balance functions in children with spastic cerebral palsy	PMC7389949	1,864
24	A review on neonatal acute respiratory distress syndrome	PMC7389165	1,834
25	Effect of advanced maternal age on birth defects and postnatal complications of neonates	PMC7389863	1,799

### Word frequency analysis

We conducted word frequency analysis using R software (version 4.1.3). By summarizing and analyzing all the analyzed articles’ MeSH keywords in PubMed, we used the dplyr package (version 1.1.2) for word frequency statistics and ranking, calculating the occurrence frequency of these keywords in the top articles for each year and the total readership of these articles.

### Co-word clustering analysis

We used VOSviewer (version 1.6.20) for co-word analysis to extract the main keywords of the high-readership articles and cluster them based on their co-occurrence frequency in the literature. All analyzed articles were imported into VOSviewer in PubMed bibliographic data format, with the minimum co-word occurrence frequency threshold set to 1 and the minimum cluster size set to 10. Other parameters were set to default, generating a keyword co-occurrence network and clustering analysis map.

## Results

### Evaluation of annual keyword readership patterns

This study analyzed the primary keywords of the top 25 high-readership articles each year from 2021 to 2023, examining the yearly keyword readership trends and visualizing the research hotspots.

### 2021 high-frequency keyword analysis

As shown in Table 4, “COVID-19” and related themes had the highest readership throughout the year, indicating that the primary research focus in 2021 was the COVID-19 pandemic. Related themes included the epidemiological dynamics of the pandemic, virus testing, treatment, and societal and economic impacts. In addition, primary research evaluated vaccine development including effectiveness, safety andstrategies for implementation.

**Table 4:** Read frequency for articles corresponding to the top 50 popular keywords from 2021 to 2023.

No.	2021		2022		2023	
	Keywords	Read frequency	Keywords	Read frequency	Keywords	Read frequency
1	COVID-19	30,510	COVID-19	54,224	Influenza, human	25,583
2	SARS-CoV-2	30,510	SARS-CoV-2	54,224	Influenza A virus	25,583
3	Betacoronavirus beta	16,787	Pandemics	29,518	Fever	22,978
4	Coronavirus infections	16,787	Betacoronavirus beta	22,714	Nucleic acids	22,978
5	Pneumonia, viral	16,787	Coronavirus infections	22,714	Time factors	22,978
6	Pandemics	15,803	Pneumonia, viral	22,714	Virus shedding	12,044
7	COVID-19 vaccines	13,723	Fever	13,226	Penis	12,044
8	Vaccines	13,723	COVID-19 vaccines	8,931	Sexual maturation	11,920
9	Retrospective studies	8,314	Vaccines	8,931	Risk factors	11,920
10	Penis	8,314	Mucocutaneous lymph node syndrome	8,690	COVID-19	8,510
11	Sexual maturation	8,314	Penis	7,656	SARS-CoV-2	8,510
12	Kawasaki disease	6,439	Sexual maturation	7,656	Pregnancy	7,989
13	Infant, premature	5,759	Nervous system diseases	6,804	Adenoviridae infections	7,860
14	Alanine transaminase	5,688	Risk factors	6,588	Pneumonia, viral	7,152
15	Asymptomatic diseases	5,688	Suicidal ideation	6,588	Behavior therapy	6,962
16	COVID-19 testing	5,688	Influenza, human	6,239	Tic disorders	6,962
17	Clinical laboratory techniques	5,688	Intubation, intratracheal	5,002	Tourette syndrome	6,962
18	Contact tracing	5,688	Lung	5,002	Infant, premature	6,412
19	Cough	5,688	Lung ventilation	5,002	Premature birth	5,946
20	Critical illness	5,688	Thoracic surgical procedures	5,002	Bronchoalveolar lavage fluid	5,359
21	Fever	5,688	Behavior therapy	4,072	*Mycoplasma pneumoniae*	5,359
22	Tomography, X-ray computed	5,688	Tic disorders	4,072	Pneumonia, Mycoplasma	5,359
23	Treatment outcome	5,688	Tourette syndrome	4,072	Suicidal ideation	4,788
24	Cerebral palsy	3,650	Alanine transaminase	3,815	Depression	4,788
25	Behavior therapy	3,157	Asymptomatic diseases	3,815	Parent-child relations	4,788
26	Tic disorders	3,157	COVID-19 testing	3,815	Parenting	4,788
27	Tourette syndrome	3,157	Clinical laboratory techniques	3,815	Betacoronavirus	4,651
28	Infant, newborn, diseases	3,005	Contact tracing	3,815	Coronavirus infections	4,651
29	Pregnancy	2,937	Cough	3,815	Mucocutaneous lymph node syndrome	4,651
30	Enteral nutrition	2,685	Critical illness	3,815	Pandemics	4,651
31	Extremities	2,304	Tomography, X-ray computed	3,815	Logistic models	4,544
32	Movement	2,304	Treatment outcome	3,815	Basal ganglia	4,145
33	virtual reality exposure therapy	2,304	Influenza A virus	3,554	Basal ganglia diseases	4,145
34	Dyspnea	1,854	Nucleic acids	3,554	Brain	4,145
35	Extracorporeal membrane oxygenation	1,854	Time factors	3,554	Iron	4,145
36	Pulmonary surfactants	1,854	Virus shedding	3,554	Iron metabolism disorders	4,145
37	Respiratory distress syndrome, newborn	1,636	Depression	3,478	Muscular atrophy, spinal	3,894
38	Child development	1,636	Parent-child relations	3,478	COVID-19 vaccines	3,859
39	Infant nutritional	1,636	Parenting	3,478	Vaccines	3,859
40	Nutritional requirements	1,625	Cerebral hemorrhage	3,452	Enterocolitis, necrotizing	3,298
41	Parenteral nutrition	1,625	Cesarean section	3,452	Child development	3,114
42	Inflammatory bowel diseases	1,625	Congenital abnormalities	3,452	Follow-up studies	3,114
43	Nutritional support	1,625	Logistic models	3,452	Gestational age	3,114
44	Stem cell transplantation	1,625	Maternal age	3,452	Attention deficit disorder with hyperactivity	2,962
45	Enterocolitis, necrotizing	1,571	Pregnancy	3,452	Autism spectrum disorder	2,962
46	Biomedical research	1,530	Pregnancy complications	3,452	Neurodevelopmental disorders	2,962
47	Neutropenia	1,539	Basal ganglia	3,438	Prospective studies	2,832
48	Urticaria	1,525	Basal ganglia diseases	3,438	Pregnancy outcome	2,832
49	Pregnancy complications	1,503	Brain	3,438	Tobacco smoke pollution	2,819
50	Streptococcal infections	1,503	Iron	3,438	Respiratory tract infections	2,819

Pediatric and adolescent neurological diseases and behavioral development were significant areas of interest for researchers. Keywords related to “cerebral palsy,” “behavioral therapy,” and “tic disorder” had readerships exceeding 3,000 times.

The management and treatment of diseases in preterm infants and newborns were also focal points. Keywords related to “enteral nutrition,” “parenteral nutrition,” and “nutritional support” in articles about preterm infants and newborns had high readerships, indicating researchers’ attention to the importance of nutrition in the treatment and health maintenance of preterm infants and newborns. Neonatal respiratory diseases were also a hot topic.

**Figure 1:**
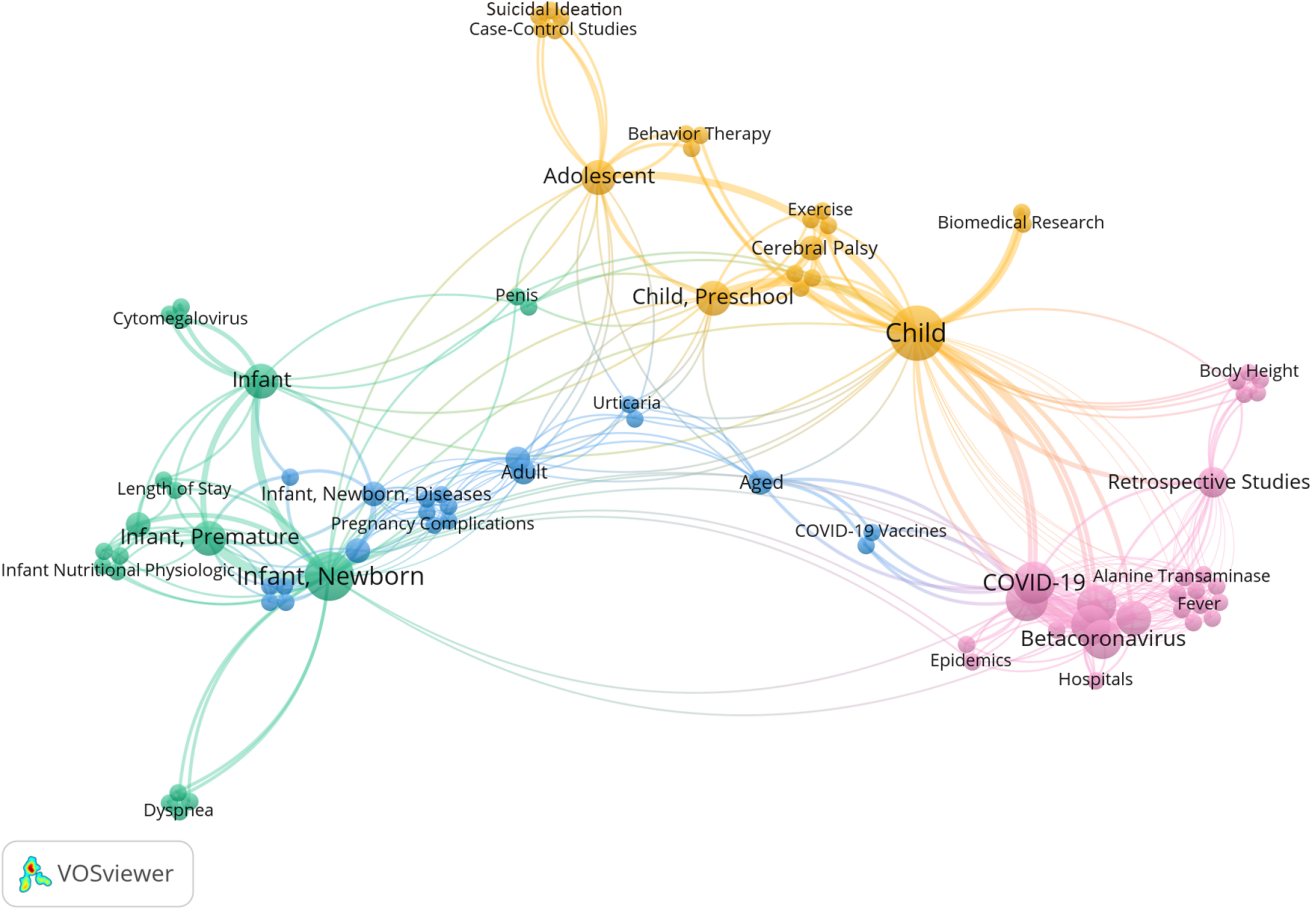
2021 co-occurrence network and cluster analysis diagram of hot keywords. From the graph, it can be observed that there were mainly four research directions: COVID-19 (in violet-red); pediatric and adolescent neurological diseases and behavioral development (in orange); preterm-related issues (in blue-green); neonatal diseases (in sky-blue).

Using VOSviewer to construct a keyword co-occurrence network and clustering map, the results were consistent with the descriptive keyword analysis mentioned above (Figure 1).

### 2022 high-frequency keyword analysis

As shown in Table 4, COVID-19 continued to be a hot topic in 2022: articles with keywords like “COVID-19” and “SARS-CoV-2” had the highest readership, indicating that COVID-19 remained a major focus in 2022. This included the development of the pandemic, virological research, epidemiological investigations, vaccine research and vaccination, and global public health responses. Influenza-related issues also gradually attracted attention.

Articles focusing on mental health and behavioral disorders in children and adolescents continued to garner high readership in 2022. Keywords like “suicidal ideation” and “depression” had readerships exceeding 6,000 and 3,000 times, respectively, highlighting the importance of research on children's mental health and behavioral disorders.

Pediatric neurological diseases and neurodevelopment continued to be highly focused research areas. This included diseases of the nervous system and related behavioral and developmental issues in children, such as autism spectrum disorder and attention deficit hyperactivity disorder.

Keywords like “cesarean section” and “pregnancy complications” also had high readerships, indicating high researcher interest in neonatal and perinatal medicine.

**Figure 2:**
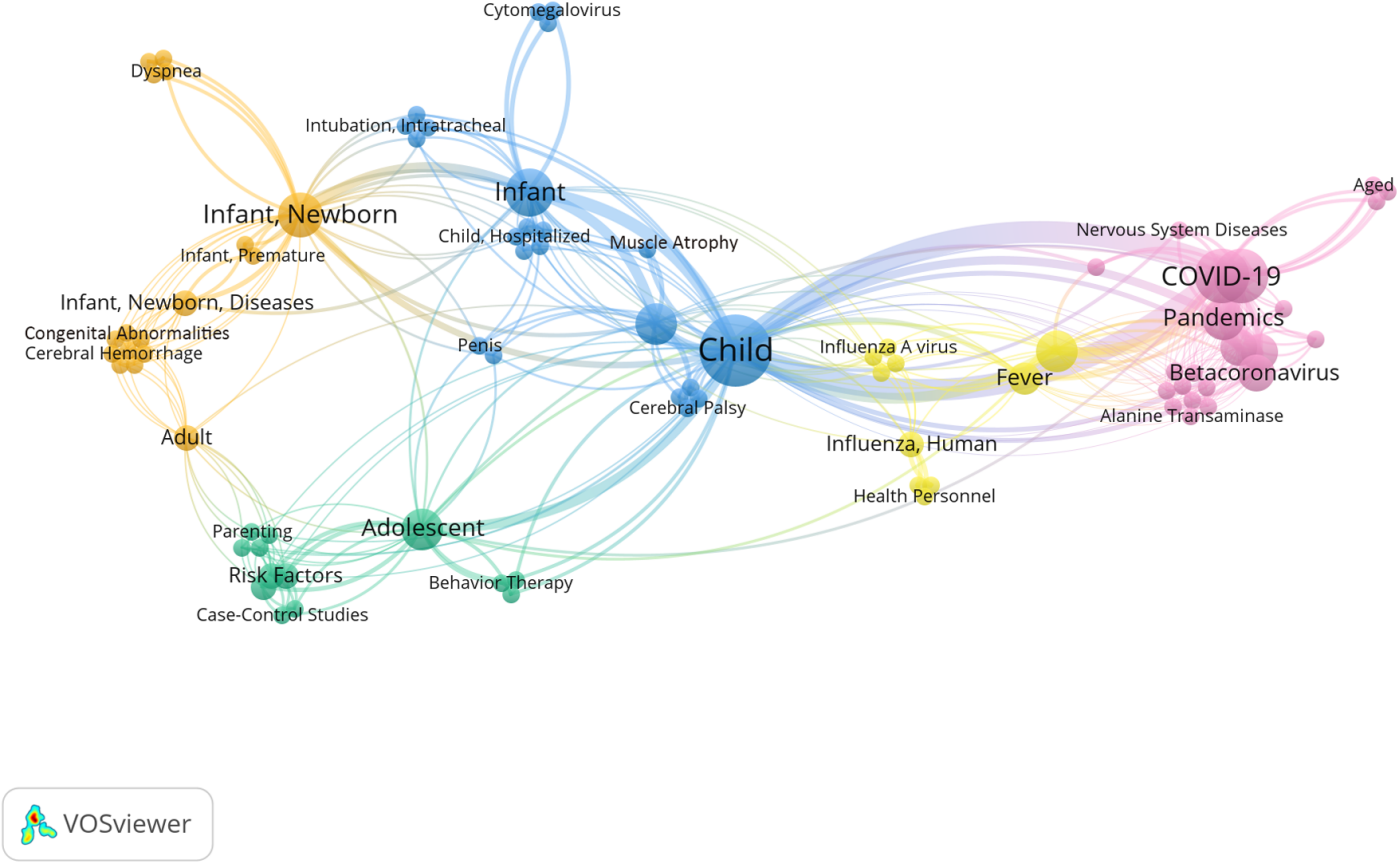
2022 co-occurrence network and cluster analysis diagram of hot keywords. From the colors and clustering of the keywords in the diagram, it can be observed that there were mainly five research directions: COVID-19 (in violet-red); influenza (in yellow); adolescent mental and behavioral health (in blue-green); pediatric neurological disorders (in sky-blue); neonatal diseases (in orange).”

Using VOSviewer to construct a keyword co-occurrence network and clustering map, the results were consistent with the descriptive keyword analysis mentioned above (Figure 2).

### 2023 high-frequency keyword analysis

As shown in Table 4, articles related to “influenza” had the highest readership in 2023, indicating that the primary focus in 2023 had shifted to influenza rather than COVID-19. However, COVID-19 still garnered significant attention, particularly regarding its long-term impacts, new variants, vaccines, and treatments.

Articles related to “risk factor research” had high readerships in 2023, indicating strong interest in disease prevention and management of risk factors in children.

In 2023, articles on “pregnancy-related research” and “preterm infant issues” also had high readerships, showing that pregnancy health, complications, and outcomes, as well as the health and management of preterm infants, were research hotspots.

Furthermore, attention to mental health issues in children and adolescents remained high in 2023, indicating it is a continuously interesting area for researchers.

In addition, in 2023, researchers showed greater attention to pediatric neurological diseases and neurodevelopment than in the previous two years. Keywords such as “tic disorder” and “Tourette syndrome” had higher readerships, and keywords related to neurodevelopmental conditions like “attention deficit hyperactivity disorder” entered the top 50 high-frequency keywords for the year.

**Figure 3:**
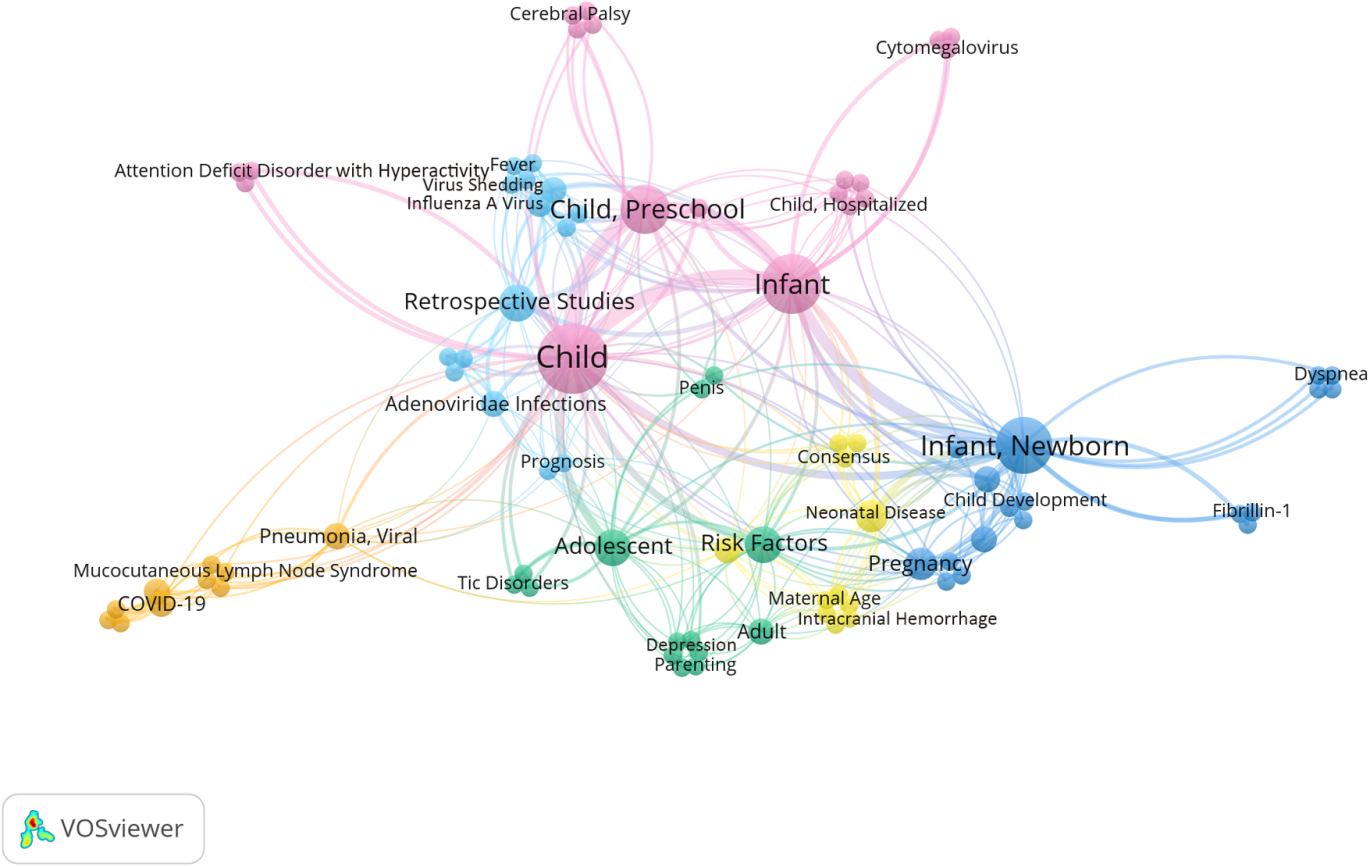
2023 co-occurrence network and cluster analysis diagram of hot keywords. From the colors and clustering of the keywords in the diagram, it can be observed that there were mainly six research directions: influenza (in sky-blue); pediatric neurological disorders and neurobehavioral development (in violet-red); COVID-19 (in orange); preterm-related issues (in blue); neonatal diseases (in yellow); adolescent mental and psychological health (in blue-green).

Using VOSviewer to construct a keyword co-occurrence network and clustering map, the results were consistent with the descriptive keyword analysis mentioned above (Figure 3).

### Overall descriptive analysis of high-frequency keywords from 2021 to 2023

From the above keyword analysis, the overall trends in readership hotspots on pediatric research can be observed.

(1) COVID-19 continued to dominate readership hotspots in 2021 and 2022, spanning two years, indicating the profound impact of the COVID-19 pandemic globally and the primary focus of researchers on this topic. However, in 2023, while COVID-19 still had significant attention, influenza and other respiratory infections became the primary focus, aligning with the 2023 global focus in pediatrics. This indicates researchers’ high attention to current major public health events and hotspots in medical health, with a great demand for information on the diagnosis, treatment, prevention, and management of these diseases.

Mental health issues in children and adolescents and neurological diseases and neurodevelopment in children were consistently focused research areas in pediatrics, with increasing attention. Research on diseases in preterm infants and newborns also continued to rise, indicating urgent research needs in these areas.

## Discussion

Word frequency analysis is a text mining technique that statistically analyzes the frequency of important words in literature or texts to reveal thematic content and hotspot trends, helping researchers understand which words are most critical in the texts and how these words change over time. Additionally, this study used VOSviewer to construct keyword co-occurrence networks and clustering maps. This bibliometric visualization analysis method offers more objective, intelligent, and precise advantages, reducing human interference and accurately identifying potential research hotspots (1, 2).

Through word frequency analysis and co-word analysis, this study explored the thematic types of high-readership articles from the *CJCP* in PMC from 2021 to 2023. Among the 1,784 articles published by the *CJCP* from 2016 to 2023, excluding pediatric surgery, various subspecialties in pediatrics were covered. The study results highlighted the main research directions that international researchers focus on, including (1) current major public health events and medical hotspots, such as COVID-19 and influenza; (2) mental health issues in children and adolescents; (3) pediatric neurological diseases and neurodevelopment; (4) diseases in preterm infants and newborns. This suggests that these research directions are currently and will continue to be valued and focused areas in pediatrics.

The COVID-19 pandemic has significantly impacted children's physical and mental health. This study showed that researchers concentrated on both the direct impacts of COVID-19 on children (such as infection symptoms, transmission modes, and control measures) and indirect impacts (such as the effects of isolation measures on children's psychology and behavior). Furthermore, with the promotion of vaccine inoculation, research focuses also included the effectiveness and safety of vaccines in children and adolescents. Articles related to COVID-19 had the highest readership in PMC from the *CJCP* in 2021 and 2022. The pandemic's peak years of 2021–2022 made COVID-19 the most focused public health event for clinical and research workers. Although attention to COVID-19 remained high in 2023, respiratory infections such as influenza became the primary focus, aligning with the global clinical focus in pediatrics in 2023. This suggests that pediatric researchers can keep pace with current major public health events or medical hotspots, sharing experiences and research findings related to these events with international peers to collectively address global pediatric health challenges. In 2023, influenza emerged as a significant topic of interest in pediatric research, particularly in the aftermath of the COVID-19 pandemic, as influenza activity gradually returned to pre-pandemic levels (3). The American Academy of Pediatrics recommends routine annual influenza vaccination for all children without medical contraindications starting at six months of age, along with the use of antiviral medications as essential strategies to mitigate influenza's impact on pediatric populations (4). Data from the 2022–2023 influenza season revealed notably high hospitalization rates for influenza among children, especially those with underlying health conditions, underscoring the severity and complexity of influenza's effects in this demographic (5). Moreover, the co-circulation of influenza with other respiratory viruses contributed to increased morbidity, highlighting the necessity for timely vaccination and treatment (3). However, many affected children were not adequately vaccinated or treated, leading to severe outcomes (5). Overall, these findings emphasize the critical importance of preventive measures and effective management strategies to protect children from influenza and its complications.

This study showed that mental health issues in children and adolescents are highly focused on by researchers worldwide, which may be due to the following reasons: (1) The rapid changes in modern social environments have increased the pressures and challenges faced by children and adolescents. Changes in family structures, school education and social competition pressures, and the widespread use of social media and the internet profoundly impact adolescents’ mental health (6–9). These factors lead to higher incidences of anxiety, depression, and behavioral problems among children and adolescents. National data from the United States in 2019 showed that among children aged 3–17, 7.1% had anxiety problems, 7.4% had behavioral problems, and 3.2% had depression (10). Meta-analysis indicated a prevalence rate of 15.4% for depressive symptoms among children and adolescents in China (11). In Hunan Province, China, the detection rates of depressive symptoms, generalized anxiety, and comorbidity of the two among middle school students in the past two weeks were 15.3%, 11.7%, and 9.0%, respectively (12). (2) Untreated mental health issues significantly affect individuals’ quality of life and impose substantial socio-economic burdens, including direct medical costs, education and productivity losses, and increased social services demand. Investing in mental health prevention and early intervention for children and adolescents can reduce long-term social and economic burdens from public health and economic perspectives.

This study indicated that pediatric neurological diseases and neurodevelopmental issues are key research areas for pediatric researchers worldwide, possibly due to the following factors: (1) Neurological diseases and neurodevelopmental disorders often have profound impacts on children's entire lives. For example, autism spectrum disorder, attention deficit hyperactivity disorder, and epilepsy not only affect children's learning and social abilities but may also persist throughout life. Early neurodevelopmental abnormalities can lead to long-term cognitive, behavioral, and social dysfunctions. Therefore, research on these diseases can improve children's short-term quality of life and reduce the long-term health burden on society. (2) Pediatric neurodevelopmental issues involve complex genetic and environmental factors, interacting to influence normal neurodevelopment. Advances in gene sequencing technology and neurobiological research now allow scientists to delve deeper into these complex biological mechanisms. These studies are crucial for developing new diagnostic methods and treatment strategies. For example, studies have shown that autism spectrum disorder is a global pediatric research hotspot (13, 14). (3) Early diagnosis and timely intervention can significantly improve prognosis for many neurological diseases. Research focuses include developing and improving early screening tools, such as using neuroimaging, biomarkers, and behavioral assessments to identify at-risk children. In addition, early intervention strategies for specific neurodevelopmental disorders, such as behavioral therapy and medication, are continuously being developed and optimized to improve patient prognosis and quality of life (15, 16). Recent systematic reviews have identified promising therapeutic strategies to improve outcomes for patients with neurological diseases. Non-pharmacological approaches—such as sensory stimulation, music therapy, virtual reality, transcranial direct current stimulation, and transcranial magnetic stimulation—show innovative potential for stimulating recovery in patients with severe brain injuries (17). Additionally, nerve growth factor has emerged as a significant therapeutic option for pediatric neurological diseases due to its ability to promote neuronal survival, repair, and plasticity. Innovative delivery methods, including intranasal administration and nanotechnology-based approaches, are being explored to enhance the effectiveness of nerve growth factor while minimizing its side effects, underscoring its potential in treating conditions like hypoxic-ischemic encephalopathy and traumatic brain injury in children (18).

This study also showed that diseases in preterm infants and newborns are key focus areas for researchers, possibly due to the following factors: (1) Preterm birth is a global critical issue, with the World Health Organization establishing November 17th as World Prematurity Day. Approximately 15 million babies are born prematurely worldwide each year, with a preterm birth rate of about 11%, and deaths due to preterm birth account for 35% of global neonatal deaths (19). Preterm infants face significant health challenges, including low vitality, immature immune systems, and high risks of chronic health issues. With increased survival rates of preterm infants, effective management of preterm sequelae and improving the quality of life of preterm infants have become important research areas. (2) Neonatal period health issues include genetic diseases, metabolic disorders, respiratory distress syndrome, and other diseases. Research in these areas aims to reduce infant mortality and improve the quality of life of surviving infants through more effective diagnosis, prevention, and treatment methods. A study based on keyword clustering analysis also found “newborns” to be a global pediatric research hotspot (14). (3) The rapid development of medical technology, particularly in genetic diagnosis, biomarkers, and monitoring equipment innovations, provides unprecedented opportunities for early diagnosis and treatment of neonatal diseases. Additionally, with social development and progress, there is increasing emphasis on the health of preterm infants and newborns. Governments and non-governmental organizations are investing more in maternal and child health projects to reduce infant mortality and improve birth quality. Research on diseases in preterm infants and newborns has long-term impacts on social and economic development.

In conclusion, this study revealed research hotspots and trends in pediatrics through word frequency analysis and co-word analysis, providing pediatric researchers with a valuable perspective to understand and grasp the development dynamics and future directions in the field of pediatrics. By exploring these hotspots, we hope to promote innovation and development in pediatrics, significantly improving children's physical and mental health and increasing international attention to researchers’ scientific achievements. While this study provides valuable insights into high readership articles within the *CJCP*, it is important to acknowledge that focusing exclusively on a single journal may limit the generalizability of our findings. The trends identified here may reflect specific interests and practices prevalent within the *CJCP* readership and may not fully capture the broader landscape of pediatric research. To gain a more comprehensive understanding of research hotspots and trends in pediatrics, future studies should consider incorporating articles from multiple journals.

## Data Availability

The original contributions presented in the study are included in the article/Supplementary Material, further inquiries can be directed to the corresponding author.

## References

[1] Xia L Li HW. Visualization analysis of highly cited and highly downloaded papers for medical journal topic selection strategy. Chin J Med Libr Inf Sci. (2021) 30:65–75. 10.3969/j.issn.1671-3982.2021.05.010

[2] Li XJ Tang DR Ma J Liu ZH Chen JY. Bibliometric analysis of the transfusion medicine research field based on PubMed database. Chin J Blood Transfus. (2020) 33(2):132–5. 10.13303/j.cjbt.issn.1004-549x.2020.02.012

[3] Merișescu MM Luminos ML Pavelescu C Jugulete G. Clinical features and outcomes of the association of co-infections in children with laboratory-confirmed influenza during the 2022–2023 season: a Romanian perspective. Viruses. (2023) 15:2035. 10.3390/v15102035 37896811 PMC10611070

[4] COMMITTEE ON INFECTIOUS DISEASES. Recommendations for prevention and control of influenza in children, 2022–2023. Pediatrics. (2022) 150:e2022059274. 10.1542/peds.2022-059274 36065749

[5] White EB O'Halloran A Sundaresan D Gilmer M Threlkel R Colón A High influenza incidence and disease severity among children and adolescents aged <18 years—United States, 2022–23 season. MMWR Morb Mortal Wkly Rep. (2023) 72:1108–14. 10.15585/mmwr.mm7241a2 37824430 PMC10578954

[6] Cong EC Cai YY Wang Y Wu Y. Association between adolescent depression and suicidal ideation and parental rearing styles. Chin J Contemp Pediatr. (2021) 23:938–43. 10.7499/j.issn.1008-8830.2105124 34535210 PMC8480172

[7] Huang XX Li YT Chen JH Ma JJ Cong EZ Xu YF. Impact of family structure on adolescent depression and anxiety symptoms: the mediating role of emotional neglect. Chin J Contemp Pediatr. (2023) 25:80–5. 10.7499/j.issn.1008-8830.2208058 36655668 PMC9893832

[8] Wang J. The relationship between parental rearing styles and anxiety and depression in elementary school students: the mediating role of academic pressure (Dissertation/master’s thesis). Tangshan: North China University of Science and Technology. (2023).

[9] Chen T Luo YL Hu HX Song XG Chen FH Fang XY Association of screen time and behavior types with anxiety and depression among middle and elementary school students in Jiangxi Province. Chin J School Health. (2024) 45:73–7. 10.16835/j.cnki.1000-9817.2024077

[10] Ghandour RM Sherman LJ Vladutiu CJ Ali MM Lynch SE Bitsko RH Prevalence and treatment of depression, anxiety, and conduct problems in US children. J Pediatr. (2019) 206:256–267.e3. 10.1016/j.jpeds.2018.09.021 30322701 PMC6673640

[11] Li JL Chen X Zhao CH Xu Y. Meta-analysis of the prevalence of depressive symptoms in Chinese children and adolescents. Chin J Child Health Care. (2016) 24:295–8. 10.11852/zgetbjzz2016-24-03-22

[12] Liu RJ Peng WJ Zheng MX Cheng SJ Hu M. The current situation and epidemiological characteristics of depression and anxiety among middle school students in Hunan Province. Chin J Clin Psychol. (2023) 31:816–20. 10.16128/j.cnki.1005-3611.2023.04.010

[13] Fan YF Li JH Shan WH Shen YM Lian ZZ Zang H Analysis of global research hotpots in the field of pediatrics based on essential science indicators and its revelation. Chin J Appl Clin Pediatr. (2022) 37:1756–60. 10.3760/cma.j.cn101070-20220927-01121

[14] Lu L Guo YL Dili R. ESI-based pedagogical high-cited papers. Med Inform. (2019) 32:1–4. 10.3969/j.issn.1006-1959.2019.22.001

[15] Chen YZ Li N Zhou KY. Observational study on the preventative treatment effects of behavioral therapy combined with fluoxetine on childhood migraine. Chin J Contemp Pediatr. (2014) 16:1105–8. 10.7499/j.issn.1008-8830.2014.11.006 25406552

[16] Di Sarno L Curatola A Cammisa I Capossela L Eftimiadi G Gatto A Non-pharmacologic approaches to neurological stimulation in patients with severe brain injuries: a systematic review. Eur Rev Med Pharmacol Sci. (2022) 18:6856–70. 10.26355/eurrev_202209_29789 36196734

[17] Capossela L Gatto A Ferretti S Di Sarno L Graglia B Massese M Multifaceted roles of nerve growth factor: a comprehensive review with a special insight into pediatric perspectives. Biology (Basel). (2024) 13:546. 10.3390/biology13070546 39056738 PMC11273967

[18] LeBlanc LA Gillis JM. Behavioral interventions for children with autism spectrum disorders. Pediatr Clin North Am. (2012) 59:147–64. 10.1016/j.pcl.2011.10.006 22284799

[19] Walani SR. Global burden of preterm birth. Int J Gynaecol Obstet. (2020) 150:31–3. 10.1002/ijgo.13195 32524596

